# An inevitable wave of prescription drug monitoring programs in the context of prescription opioids: pros, cons and tensions

**DOI:** 10.1186/2050-6511-15-46

**Published:** 2014-08-16

**Authors:** M Mofizul Islam, Ian S McRae

**Affiliations:** 1 Australian Primary Health Care Research Institute, Australian National University, Building 63, corner of Mills & Eggleston Roads, Canberra, ACTON ACT 0200, Australia

**Keywords:** Prescription drug monitoring programme, Prescription drugs, Controlled drugs, Prescription opioids

## Abstract

**Background:**

In an effort to control non-medical use and/or medical abuse of prescription drugs, particularly prescription opioids, electronic prescription drug monitoring programs (PDMP) have been introduced in North-American countries, Australia and some parts of Europe. Paradoxically, there are simultaneous pressures to increase opioid prescribing for the benefit of individual patients and to reduce it for the sake of public health, and this pressure warrants a delicate balance of appropriate therapeutic uses of these drugs with the risk of developing dependence. This article discusses pros and cons of PDMP in reducing diversion of prescription opioids, without hampering access to those medications for those with genuine needs, and highlights tensions around PDMP implementation.

**Discussion:**

PDMPs may help alleviate diversion, over-prescription and fraudulent prescribing/dispensing; prompt drug treatment referrals; avoid awkward drug urine test; and inform spatial changes in prescribing practices and help designing tailored interventions. Fear of legal retribution, privacy and data security, potential confusion about addiction and pseudo-addiction, and potential undue pressure of detecting misuse/diversion - are the major problems. There are tensions about unintended consequence of excessive regulatory enforcements, corresponding collateral damages particularly about inadequate prescribing for patients with genuine needs, and mandatory consultation requirements of PDMP.

**Summary:**

In this era of information technology PDMP is likely to flourish and remain with us for a long time. A clear standard of practice against which physicians’ care will be judged may expedite the utilisation of PDMP. In addition, adequate training on addiction and pain management along with public awareness, point-of-supply data entry from pharmacy, point-of-care real-time access to data, increasing access to addiction treatment and appropriate regulatory enforcement preferably through healthcare administration, together, may help remove barriers to PDMP use.

## Background

In an effort to control non-medical use and/or medical abuse of prescription drugs, particularly prescription opioids and benzodiazepines, prescription drug monitoring programmes (PDMP) have been introduced in North-American countries, Australia and some parts of Europe. Although the history of PDMP goes back to the early twentieth century [1] an increasing non-medical use and/or medical abuse of controlled drugs and associated deaths observed in recent times, and the computerisation of PDMP, have brought it back into focus. Ironically, there are simultaneous pressures to increase opioid prescribing for the benefit of individual patients and to reduce it for the sake of public health [2]. This dilemma puts increasing pressure on policy makers to balance appropriate therapeutic uses of these drugs with the risk of developing dependence, abuse and fatality from overdose.

Although programme specifics vary across settings, generally electronic PDMPs require retail pharmacists to enter data from prescriptions for controlled substances into a centralised electronic database. These data identify the prescriber, dispenser, and patient, as well as the drug, dose, and amount dispensed. Some PDMPs require additional information which helps pharmacists/prescribers to track and identify duplicates and stolen forms [3]. The initial purpose of PDMP was to detect and reduce diversion, abuse, and misuse of prescription medications classified as controlled substances, and to reduce associated harms, although a PDMP can of course also be used to provide information to enable physicians to offer more appropriate care. The aim of this article is to discuss, based upon the available literature, the pros and cons of PDMP in achieving the overarching goal of reducing inappropriate prescribing, particularly of opioids (while the discussion in this paper relates to other controlled substances, the paper will focus on opioids), without hampering access to essential medications for those with genuine needs.

### Pros

#### ***Reduce over prescription and doctor shopping***

In day-to-day clinical practice it may be difficult to discern a patient who legitimately suffers pain from one who is pretending to be in pain for the sake of obtaining drugs. Inappropriate prescribing of controlled substances, particularly those prescribed as pain killers, can arise where a patient pretends to have pain or amplifies pain. One dimension is to identify those who either initially through pain management or through other routes developed dependence and are using multiple medical providers in order to abuse prescription pharmaceuticals. Pain is a condition that is almost impossible to measure from laboratory tests, radiologic imaging, or physical examination, physicians rely largely on patient interviews and histories, depend on interpretation of what they see and hear [4]. PDMPs can add accuracy to providers’ clinical judgments to determine the requirements for pain management.

A PDMP will inform a physician if a patient has recently accessed opioids elsewhere. This will help to identify doctor shoppers (or prescription shoppers), or persons who have no genuine medical need. By identifying these patients, a real-time PDMP may help alleviate over-provision and potentially diversion of these pharmaceuticals. As an added benefit, prescribers may monitor the database to detect forged prescriptions or stolen prescription pads/pages [2].

#### ***Reduce fraudulent prescribing by physicians***

In addition to addressing issues of patients, PDMPs can help to identify any suspected fraudulent prescribing or illegal activity related to the dispensing of controlled substances, and inform the professional licensing boards of these clinicians. In Florida, for example, PDMP played an important role in the improvements in its prescription drug-abuse record. In 2010, among the top 100 oxycodone purchasing physicians in the nation 90 were located in Florida. This number dropped to only 13 in 2011.

#### ***Quality of care***

A different but arguably more important aspect of the use of PDMPs is that the provision of up-to-date information about controlled substance use across all practices and physician’s access to that information can support clinical judgement and improve the quality of care. PDMP can help identify patients who are receiving multiple legitimate prescriptions but are at risk of complications from polypharmacy [2].

Health professionals’ access to a real-time PDMP may facilitate a patient-centred approach to addressing prescription drug abuse. It is more comfortable checking a PDMP report than mandating urine drug screening, which can result in disruption of the patient-physician relationship [5]. Furthermore, patients’ awareness of the PDMP database may lead them to provide information which would enhance the patient-provider relationship. PDMP may help identify those at highest risk for opioid overdose and so create an opportunity for intervention when aberrant behaviour is first noted [2]. A survey in Maine, USA found PDMP helped over a third of the prescribers referring their patients to substance abuse treatment [6].

Real-time access to a patient’s prescription history increases prescriber’s confidence in prescribing. For instance, a study of real-time PDMP in an emergency department in Ohio found that after reviewing the patient’s prescription history clinicians changed their opioid prescription in 41% of cases, of which more than a third (39%) received higher doses than initially planned [7].

#### ***Geographic trends of use***

PDMPs contain a wealth of information about the demographic trends and types of prescription drug use, poisoning and overdose which may be analysed to reveal changes in prescribing practices and patterns that are shaping the evolving trends. Data from prescription monitoring programmes may also be used to identify geographic areas with high rates of opioid misuse, allowing the introduction of focused interventions in those communities. Such an approach could improve the reach of existing preventive and/or associated programmes [8].

### Cons and tensions

#### ***Physician concerns***

There is concern among some physicians who for valid reasons are relatively high prescribers that if they are identified by a PDMP they may be seen to be prescribing inappropriately. While most physicians would be expected to support interventions to prevent fraudulent prescribing, high profile criminal prosecutions of physicians prescribing large amounts of opioids in USA [9,10] could make the physicians reluctant to prescribe controlled substances for fear of legal retribution (“chilling effect”) [1]. Although formal research on the chilling effect is rare, surveys indicate that some prescribers underutilise controlled substances due to fear of legal repercussions [11-13]. There is greater perceived legal risk for prescribing/dispensing too much pain medication than for prescribing/dispensing too little pain medication [14].

The chilling effect could also lead to increased prescribing of alternate medications (substitution effect), even if they are inferior in terms of effectiveness or have greater side effects. Many of these alternate medications are uncontrolled medications – as was noted when benzodiazepines were added to in New York’s paper-based prescription monitoring registry in 1989 [15]. Another example is increased prescribing of Schedule III opioids in California after it removed the triplicate prescription system in order to reduce prescribing of Schedule II drugs [9].

#### ***Pseudo addicts***

A PDMP may deter legitimate prescribing for a patient with a history of receiving pain medication from several physicians. Such patients may be “pseudo-addicts” whose pain has not been controlled by sub-therapeutic analgesics doses and who is genuinely seeking relief of pain, not support of an addiction [16].

#### ***Patient concerns about refusal to prescription and consequences***

Like prescribers, patients may fear coming under scrutiny from law-enforcement if they use medications monitored by the PDMP. Patients may worry about the additional cost of more frequent office visits if prescribers become more cautious about writing prescriptions with refills [15]. Patients who are questioned about substance use and then excluded from an expected treatment may feel embarrassed or abandoned. PDMP can produce pressure on physicians/pharmacists to detect and respond to suspected misuse/diversion and this may negatively impact on service rapport and trust. Patients who have a history (past or present) of opioid dependency are at risk of not obtaining the treatment for valid conditions [17]. These can result in people not returning to the physicians for on-going care. There is concern that this refusal could eventually push some patients into the illicit market [18]. For instance, the opioid abuse crackdown in Florida has been followed by an indication of increased use of heroin, and overdose presentation in the emergency department [19].

#### ***Wrongful categorisation as fraudulent prescribers***

Many practicing physicians have little if any formal training to enable them to identify prescription drug abuse or recognise the warning signs of drug diversion. In USA over 40% of primary care physicians report difficulty in discussing the possibility of prescription medication abuse with patients and over 90% fail to detect symptoms of substance abuse [20]. Amid these knowledge-gaps and difficulties PDMPs may wrongfully suspect and categorise some conscientious and caring physicians as fraudulent prescribers when they are actually prescribing in good faith but lack training.

#### ***Privacy***

There is discomfort among physicians and patients that PDMP means that a medical consultation is no longer a private affair, and raises concerns about maintaining patient/provider privacy, confidentiality and data security. Data entry from a huge network of pharmacies and staff access to the database make the system susceptible to unscrupulous use and data leaking. Already there have been some incidences of privacy breaches. The Florida database which leaked the personal information of thousands of patients who were reportedly not all relevant to county prosecutors investigating a criminal case, is one such example [21]. Privacy concern may also lead some patients to avoid or to postpone needed medication for fear of being labelled as drug addicts. Both American Medical Association and American Society of Addiction Medicine stress the need to treat PDMP information just as well as, if not better than, any other medical record [22,23].

#### ***Law-enforcement or healthcare?***

Recognition of prescription drug abuse has evoked many regulatory and legislative actions, and in some settings healthcare policy is increasingly influenced by law-enforcement agencies. By their nature, law-enforcement agencies will focus on the abuse side of the equation, without always considering any detrimental effect from inadequate prescribing. The abuse of prescription drugs is a multifaceted problem and needs collaboration of various stakeholders. Abuse of these drugs is more of a public health issue than anything else, and requires a public health focus – as opposed to a strictly law-enforcement focus [24]. However, in some settings with origins in law-enforcement, PDMPs are seen as a tool of the police rather than an important component of patient safety. With that police tool in mind many doctors see PDMP as a thinly veiled means of police looking over their shoulders and have fear of coming under scrutiny by law enforcement agencies [25]. Such a scrutiny to doctors, who for legitimate reasons need to prescribe large amount of opioids even if these are appropriate, is definitely a nuisance. The American Medical Association supports PDMP programmes being housed with a state agency whose primary purpose is health care quality and safety.

#### ***Balance between opioid dependence and pain management***

It is recognised that dichotomisation of users into ‘pain patients’ and ‘illicit users’ is an oversimplification of a complex issue, and there is overlap between these two groups [26]. The size of the population who experience chronic pain is quite large; in USA alone it is approximately 100 million [27]. Opioid dependence has been estimated to affect over one-third of patients with chronic pain [28]. While PDMP may help physicians in identifying and treating patients with dependence, there is a huge challenge in treating concurrent opioid dependence and pain, and finding the balance between minimising risks and negative consequences of opioid use whilst not reducing effective pain treatment [29]. Referring patients for dependence treatment has also major impediments as treatment systems are largely developed for illicit drug users, and are not suitable for a other groups [18].

#### ***Mandatory use of PDMP, time demands and patient satisfaction***

PDMP consultation may create additional time pressure on physicians. Identification of potential abuse warrants a series of responses including counselling and referral for substance abuse treatment – which are also time demanding. Amid these pressures, encouraging physicians to use PDMP databases in settings where it is voluntary has been a challenge, part of which relates to making the databases more convenient for physicians that include real-time data provision, easy recovery of forgotten passwords [6] and easy navigation to the web portal [2]. There is proposal to apply mandatory consultation requirements in certain cases, for instance, when the physician needs to prescribe certain quantity of medication or to a certain group of patients [30]. However, this may lead some providers to discontinue or further cut back on controlled substance prescribing. There is also tension around PDMP-driven patient dissatisfaction. Doctors who refuse to prescribe opioids to certain patients out of concern about abuse are likely to get a poor survey-ratings, which can affect physicians’ reimbursement and job security [4] (Table 1).

**Table 1:** Potential benefits, unintended consequences and tensions around PDMP

**Pros**	**Cons and tensions**
▪ Informed and safe prescribing for patients.	▪ Patient may not receive sufficient medications due to physicians’ fear of legal retribution (“chilling effect”).
▪ An appropriately programed real-time PDMP is likely to reduce prescription drug diversion, doctor shopping, and related casualties.	▪ Chilling effect may influence increased prescribing of inappropriate or inadequate alternate medications (substitution effect).
▪ Reduction of overprescribing by the physicians.	▪ May deter legitimate prescribing by creating confusion between the concepts of addiction and pseudo-addiction, and in treating patients with opioid dependence and pain.
▪ Reduced risk of complications from polypharmacy.	▪ Patients may fear of coming under scrutiny by law enforcement agencies and be deprived from medications.
▪ Help avoiding awkward patient confrontation such as urine drug screening, and promote a more patient-centered approach to quality use of opioids.	▪ PDMP-induced reduction of prescription opioids may increase crime particularly among illicit drug users, and push some pain patients into the illicit market.
▪ Help monitor and detect forged prescription or stolen prescription pad/page.	▪ Fear among the physicians of being categorised as fraudulent prescribers when they are actually prescribing in good faith but lack training.
▪ Help reducing fraudulent prescribing and inform the professional licensing boards about inappropriate prescribing/dispensing.	▪ Privacy concern and data security.
▪ May reveal changes in prescribing practices and patterns, and spatial information in small geographical area may inform tailored intervention.	▪ May negatively impact on service rapport and trust.

## Discussion

While there are a range of advantages and disadvantages to PDMP systems from a public health perspective, given the perceived benefits of these systems to law enforcement, to health regulators, and to public and private funders of health care, in conjunction with the trends to various forms of electronic patient records which facilitate the extraction of the necessary data, it seems likely that PDMP will remain and probably expand over time. In these circumstances the public health endeavour will be to find a balance which ensures the health gains from access to this information more than offsets any health losses from physician or patient reaction to the systems.

The reduction of substance abuse and the promotion of effective pain management are both equally important objective. However, a very small proportion of patients are involved in drug diversion/abuse; according to a recent study only 0.7% of patients obtained their prescriptions from a suspiciously large number of different prescribers [31]. Thus in evaluating a PDMP one must also consider how many patients with genuine needs of pain medications have not been prescribed – the literature consistently shows multiple-copy prescription monitoring programmes reduced access to pain medication for patients with genuine needs [32].

Clearly, abuse of prescription opioids is a multidimensional problem. While there are no simple solutions for effective prevention, some measures could be taken immediately. One such measure is to offer up-do-date information to that section of providers who have insufficient knowledge about PDMP and prescription drug addiction. This issue can be addressed through medical education curriculum and continuing programs for the physicians and pharmacists. Physicians’ willingness to fill their knowledge-gap about prescription opioids and PDMP are well recorded in the literature [20,33,34]. Another measure is to educate patients about prescription opioids, their appropriate use, potential risks and proper disposal techniques, and the necessity and importance of PDMP in prescribing opioids. Physicians are better placed to do that comfortably.

Improving the existing PDMPs to real-time programs is another highly recommended measure. Physicians and pharmacists who currently use PDMP or are willing to use this program often report its lack of real-time data provision. Unfortunately only in few settings authorities could ensure a real-time data transmission at the point of dispensing. Currently, the most common time frame for data transmission is ranging from monthly to daily [30]. In fact, as demonstrated in British Columbia, the usefulness of a PDMP, to a large extent, is attributable to the speed of its data collection [35].

A major driver of the risk of use of PDMPs leading to under-prescribing is the threat of patients or physicians being pursued by law-enforcement officials. These concerns will largely be mitigated if clear standards of practice against which physicians’ care will be judged are available, and if enforcement is seen as appropriate. The first round of response to any perceived misuse should be undertaken by healthcare administrations and these concerns should be only passed on to law-enforcement authorities after investigation by the healthcare bodies. Such an approach is likely to alleviate some pressures on physicians and patients and address some concerns about privacy, although not completely. This compromise about privacy is perhaps a necessary cost to prevent unscrupulous demand, prescribing or dispensing, and highlights that the duty of care may override such privacy considerations [36] Strong data security and privacy measures must be in place and be known to be in place. Law-enforcement agencies should not have access to patient specific PDMP data unless they have an active investigation, and healthcare providers should only have access to data relevant of their patients [37].

Data regarding the overall effects of electronic PDMPs, and in particular in relation to the balance of impacts on over and underutilisation, are limited, with aggregate studies tending to show little impact, and more qualitative studies tending to show participants do believe the PDMPs have an impact on behaviour. One American study found no difference in the incidence of opioid overdose mortality between states with and without PDMPs, and PDMPs’ effect on overall consumption of opioids appeared to be minimal [3]. Surveys of prescribers, pharmacists, and law-enforcement officials who use PDMP suggest that it is helpful in curbing abuse and diversion [32]. The few other evaluations that have been conducted thus far have found that the programmes are somewhat successful at reducing diversion [1,35,38,39].

A recent study from Canada found no differences in the opioid prescribing rate when comparing provinces with and without PDMPs [40]. However, Simeone et al. found PDMPs that issue reports proactively are likely to change prescriber behaviour in a way that reduces per capita supply of prescription pain relievers/stimulants, “which in turn reduces the likelihood of abuse” [41]. Although there is a correlation between overall supply and the likelihood of misuse [42], it is unclear whether this PDMP‒induced reduced supply translates into lower levels of opioid related harms and problems or whether there could have been both an increased under-treatment of pain or both [18,43].

Given the international trends amidst an increasing concern about abuse of prescription opioids, PDMPs are likely to gain more ground and expand, and the current reluctance among a portion of physicians/pharmacists to PDMP use is likely to disappear over time. We have evidence from similar events. When the supervised dispensing of methadone was introduced in 1996 in the UK some practitioners regarded it as an intrusion upon their clinical autonomy, but in the decades since then prescribers have followed the treatment guidelines [44].

In the discourse on PDMP, one thing often overlooked is the information and communication technology breakthroughs, networking and internet that enable work to be separated from time and space. The information technology improvement is running so fast, people are being so much habituated to it that, despite its problems, PDMP will continue. However, this does not mean that everyone will have to accept it passively. PDMP needs to be shaped appropriately so that it caters the needs of providers and patients. The sooner the prescribers and dispensers shape the PDMP the way they want and embrace it, the better the outcomes will be for their patients.

## Conclusion

Despite some unintended consequences of PDMP, it is likely to flourish and remain with us for a long time. Continued improvements in information technology may work as a catalyst in PDMP proliferation. A clear standard of practice against which physicians’ care will be judged may expedite the utilisation of PDMP. In addition, adequate training on addiction and pain management along with a provision of appropriate access to PDMP, public awareness, point-of-supply data entry from pharmacy, point-of-care real-time access to data, increasing access to addiction treatment and recovery, appropriate enforcement preferably through healthcare administration, together, may accelerate the acceptance and help realise the full benefits of PDMP.

## Abbreviations

PDMP: Prescription drug monitoring programme; USA: United States of America; UK: United Kingdom.

## Competing interests

The authors declare that they have no competing interests. The conclusions and opinions expressed in this article are those of the authors and do not necessarily reflect those of their respective organisations.

## Authors’ contributions

MMI conceptualised the paper and undertook the literature review. Both MMI and ISM were involved in acquisition and analysing the results and drafting the manuscript. Both authors read and approved the final manuscript.

## Pre-publication history

The pre-publication history for this paper can be accessed here:

http://www.biomedcentral.com/2050-6511/15/46/prepub
